# PLC1 mediated Cycloastragenol-induced stomatal movement by regulating the production of NO in *Arabidopsis thaliana*

**DOI:** 10.1186/s12870-023-04555-7

**Published:** 2023-11-17

**Authors:** Juantao Kong, Rongshan Chen, Ruirui Liu, Wei Wang, Simin Wang, Jinping Zhang, Ning Yang

**Affiliations:** College of Life Science, Northwest Normal University, Lanzhou, 730070 China

**Keywords:** Cycloastragenol, Stomatal movement, Phospholipase C1, Gα-submit of the heterotrimeric G-protein, Nitric oxide

## Abstract

**Background:**

*Astragalus* grows mainly in drought areas. Cycloastragenol (CAG) is a tetracyclic triterpenoid allelochemical extracted from traditional Chinese medicine *Astragalus* root. Phospholipase C (PLC) and Gα-submit of the heterotrimeric G-protein (GPA1) are involved in many biotic or abiotic stresses. Nitric oxide (NO) is a crucial gas signal molecule in plants.

**Results:**

In this study, using the seedlings of *Arabidopsis thaliana (A. thaliana)*, the results showed that low concentrations of CAG induced stomatal closure, and high concentrations inhibited stomatal closure. 30 µmol·L^−1^ CAG significantly increased the relative expression levels of *PLC1* and *GPA1* and the activities of PLC and GTP hydrolysis. The stomatal aperture of *plc1*, *gpa1*, and *plc1/gpa1* was higher than that of WT under CAG treatment. CAG increased the fluorescence intensity of NO in guard cells. Exogenous application of c-PTIO to WT significantly induced stomatal aperture under CAG treatment. CAG significantly increased the relative expression levels of *NIA1* and *NOA1*. Mutants of *noa1*, *nia1*, and *nia2* showed that NO production was mainly from *NOA1* and *NIA1* by CAG treatment. The fluorescence intensity of NO in guard cells of *plc1*, *gpa1*, and *plc1/gpa1* was lower than WT, indicating that *PLC1* and *GPA1* were involved in the NO production in guard cells. There was no significant difference in the gene expression of *PLC1* in WT, *nia1*, and *noa1* under CAG treatment. The gene expression levels of *NIA1* and *NOA1* in *plc1*, *gpa1*, and *plc1/gpa1* were significantly lower than WT, indicating that *PLC1* and *GPA1* were positively regulating NO production by regulating the expression of *NIA1* and *NOA1* under CAG treatment.

**Conclusions:**

These results suggested that the NO accumulation was essential to induce stomatal closure under CAG treatment, and *GPA1* and *PLC1* acted upstream of NO.

**Supplementary Information:**

The online version contains supplementary material available at 10.1186/s12870-023-04555-7.

## Introduction

Allelopathy mainly refers to plants releasing allelopathic substances into the environment through leaching, volatilization, root secretion, leaf decomposition, and residual strain. These allelopathic substances inhibit or promote the growth of plants, secreting those allelopathic substances or their neighboring plants [1]. The allelochemicals are mainly generated from plant secondary metabolites, among which terpenoids are the second-largest type of allelochemicals [2], and Cycloastagenol (CAG) is a tetracyclic triterpenoid allelochemical, mainly obtained from the hydrolysis of astragaloside IV [3]. It has protective effects from cardiovascular diseases, fatty liver, abdominal aortic aneurysms, and other diseases [4]. It can activate telomerase [5], regulate immunity, and promote wound healing and hair growth. The study found that the allelopathic effect of an extract of *Astragalus strictus* root tissues on the following wild plants of Tibet: *Medicago lupulina*, *Elymus nutans*, *Oxytropis microphylla*, *Festuca ovina*, *Stipa purpurea*, *Stipa capillacea*, *Kobresia littledalei*, and *Eragrostis nigra* [6]. It has also shown that CAG can significantly affect the telomerase system and defense signaling pathways in *Arabidopsis thaliana* (*A. thaliana*) [7, 8].

A large number of phospholipases catalyze the hydrolysis of phospholipids in nature. According to the position of the phospholipid cleavage bond in the substrate, phospholipases are divided into phospholipase A1 (PLA1), phospholipase A2 (PLA2), phospholipase C (PLC), and phospholipase D (PLD) [9]. PLC, as an essential hydrolase, is related to a variety of physiological and stress-resistant functions of plants. Among them, PLC is activated and uses PIP2 as a substrate to hydrolyze to generate DAG and IP_3_ [10]. DAG is phosphorylated to generate PA. IP_3_ is phosphorylated to generate IP_6_, which mediates the release of Ca^2+^ [11]. Then, it further participates in cell growth and differentiation, hormone signal transduction, response to biological and abiotic stress, and regulation of polar growth. Lee Hunt et al. [12] found that PLC participates in the regulation process of abscisic acid (ABA) controlling stomatal closure. Under the induction of ABA, the downstream signaling molecule IP_6_ of PLC regulates the change of intracellular Ca^2+^ level, thereby reducing the intracellular turgor pressure and finally causing stomatal closure [13]. The PLC inhibitor U73122 can inhibit the stomatal closure and Ca^2+^ oscillation by ABA and reduce the expression level of PI-PLC, which also weakens the sensitivity of tobacco leaves to ABA [14]. Although PLC is involved in many plant growth and development process, it is still unclear whether PLC plays a role in plants’ response to allelopathy of CAG.

The heterotrimeric G protein consists of three subunits of α, β, and γ. It plays roles in plant seed germination, seedling growth and development, and plant reproduction-related processes [15]. It also participates in the physiological processes of plants induced by hormones, such as auxin, brassinolide, gibberellin, and abscisic acid [16, 17]. Wang et al. [18, 19] proved that ABA could inhibit stomatal opening by activating S-type anion channels, but cannot activate the anion channels of *gpa1* mutant guard cells, indicating that Gα-submit of the heterotrimeric G-protein (GPA1) is involved in the regulation of plant anion channels. In the stomatal response triggered by ExCaM, Gα can activate the Ca^2+^ channel by promoting the formation of hydrogen peroxide (H_2_O_2_) and Nitric oxide (NO) in *A. thaliana* guard cells to induce stomatal closure [20]. Zhang et al. [21] showed that PLDαl and GPA1 are involved in the stomatal closure triggered by the diterpenoid Oridonin, and GPA1 is located upstream of PLDα1. As a functional subunit of the G protein, GPA1 participates in the various plant stress responses. However, it has not yet been reported whether GPA1 participates in the process of the allelopathy of CAG or not.

NO is a vital gas signal molecule in plants, which regulates various physiological processes of plants, including seed germination [22], photomorphogenesis [23], root growth and development [24], fruit and other tissue maturities, and senescence, programmed cell death stomatal movement, various stress responses, and disease-resistant defense responses, etc. [25]. Many studies have shown that NO is essential for ABA, SA, and JA to induce stomatal closure [26, 27]. When the NO scavenger 2-(4-carboxyphenyl)-4, 4, 5, 5-tetramethylimidazoline-1-oxyl-3-oxide potassium (c-PTIO, an NO scavenger) is applied, the hormone-induced stomatal closure is inhibited.

Until now, the allelopathic effects of CAG in plants have not been reported. In this work, results analyzed the effects of CAG treatment on stomatal movement in *A. thaliana* and the roles that PLC1, GPA1, and NO play in this process. In addition, the results reveal the allelochemical effect of the triterpenoid CAG on *A. thaliana* and provide a theoretical basis for the future application of triterpenoid allelochemicals in stomata. The study will lay a foundation for agricultural production in the future.

## Results

### CAG-induced stomatal movement in *A. thaliana* wild type

**Fig. 1:**
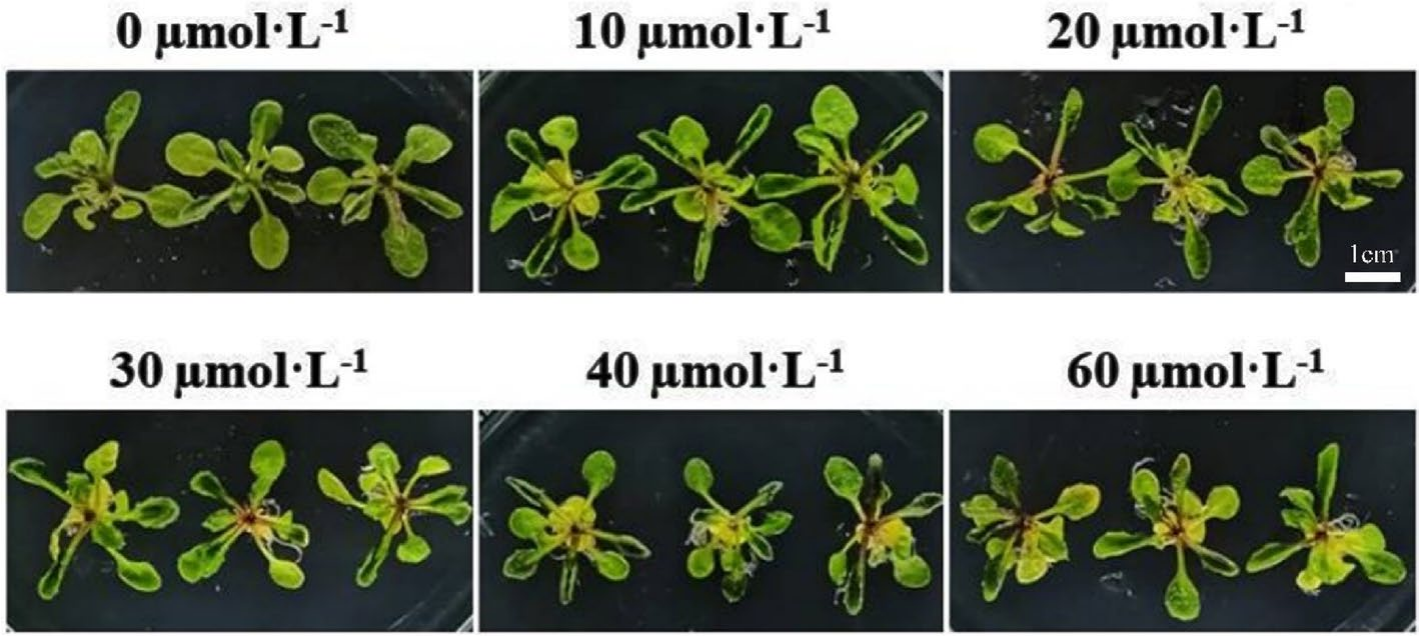
Effects of different concentrations of CAG on the growth of *A. thaliana* seedlings

The growth status of *A. thaliana* seedlings treated with different concentrations of CAG for 5 days is shown in Fig. 1. The seedlings were growing normally, and the leaves were green and spreading after 0–20 μmol·L^–1^ CAG treatment. But after 30–60 μmol·L^–1^ CAG, the leaves gradually turned yellow. These results reflected that low concentrations of CAG could promote *A. thaliana* seedling growth while high concentrations of CAG can inhibit *A. thaliana* seedling growth.

**Fig. 2:**
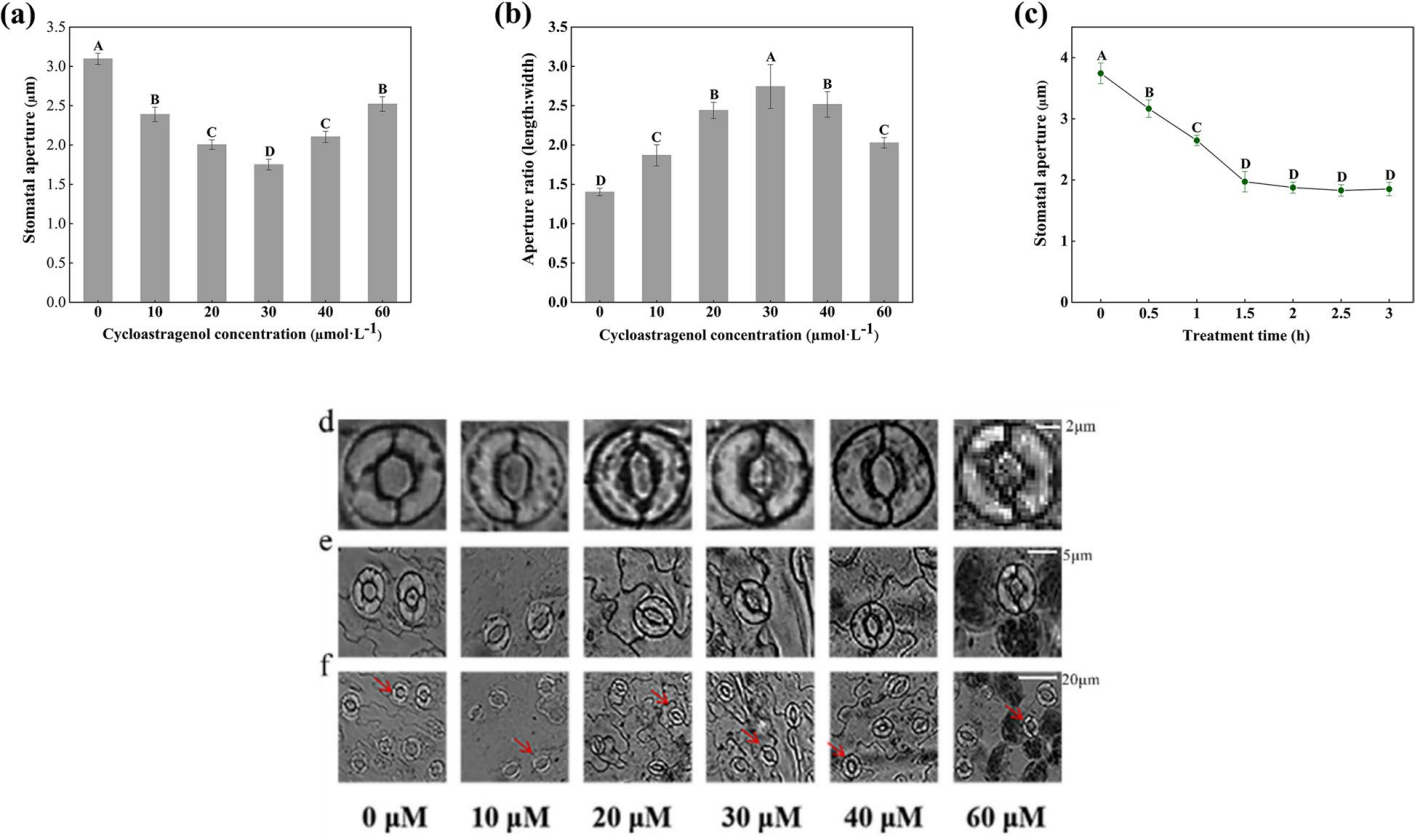
CAG-induced stomatal movement in *A. thaliana* leaves. **a** Effects of different concentrations of CAG on stomatal movement of *A. thaliana* leaves. **b** The aspect ratio of stomatal aperture. **c** Time effect of CAG-induced stomatal movement. **d** The stomates of *A. thaliana* leaves were treated with CAG at different concentrations. Bar = 2 µm. **e**, **f** A part of the stomates on the epidermis from *A. thaliana* leaves. Bar = 5 µm, Bar = 20 µm. Image f was taken at 20x, and images e and d were each magnified by 2.5x. Uppercase letters indicate differences between groups, lowercase letters indicate differences within groups. Means with different letters are significantly different at *P* < 0.05

And then to determine the effect of CAG on stomatal movement in *A. thaliana* leaves. The stomates treated with the different concentrations of CAG at different time are shown in Fig. 2. Results show that the stomatal apertures treated with 30 µmol·L^−1^ CAG was significantly lower than that of the control group (0 µmol·L^−1^ CAG). Stomatal apertures decreased significantly after 30 µmol·L^−1^ CAG treatment for 1.5 h. Therefore, 30 µmol·L^−1^ CAG treatment for 1.5 h was selected for further experiments.

### PLC1 was activated by GPA1 and then involved in CAG-induced stomatal movement

**Fig. 3:**
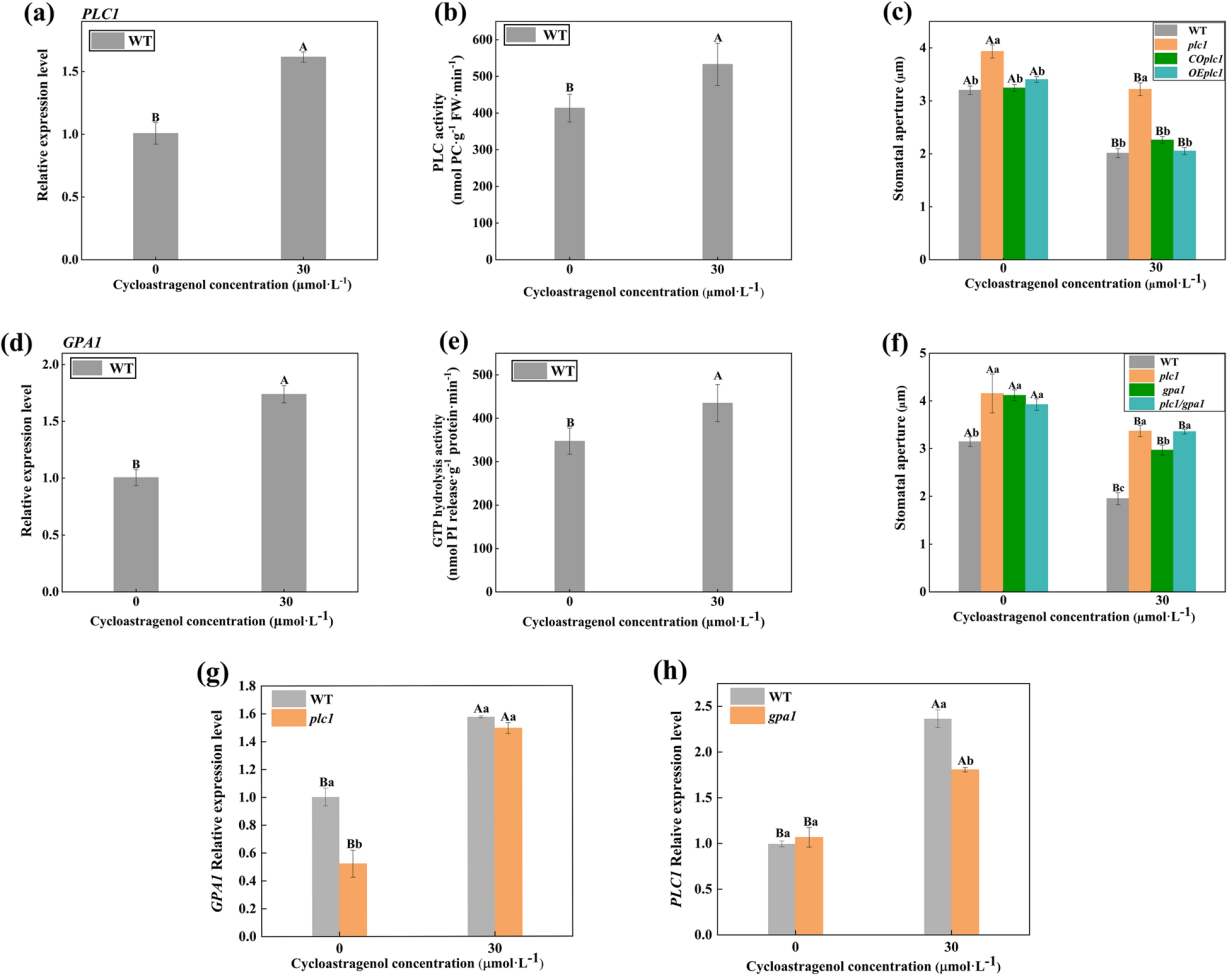
PLC1 and GPA1 were involved in the CAG-induced stomatal movement. **a** and **d** Relative expression level of PLC1 and GPA1 under the CAG treatment in WT. **b** and **e** Activities of PLC and GTP hydrolysis under the CAG treatment in WT. **c** Stomatal apertures of *A. thaliana* leaves under the CAG treatment in WT, *plc1*, *PLC1-CO*, *PLC1-OE*. **f** Stomatal apertures of *A. thaliana* leaves under the CAG treatment in WT, *plc1*, *gpa1*, *plc1/gpa1*. **g** and **h ***GPA1* and *PLC1* relative expression under the CAG. Uppercase letters indicate differences between groups, lowercase letters indicate differences within groups. Means with different letters are significantly different at *P* < 0.05

The gene-relative expression levels of *PLC1* and *GPA1* and the enzymes activity levels of PLC and GTP hydrolysis were increased markedly at 30 µmol·L^−1^ CAG treatment, indicating that PLC1 and GPA1 involved in CAG-induced stomatal movement (Fig. 3a, b, d, e). As shown in Fig. 3c, the inhibition effect of CAG on stomatal apertures of *plc1* was significantly lower than that of WT, but *COplc1*, and *OEplc1* was similar to WT, indicating that PLC1 positively regulated the stomatal closure. As shown in Fig. 3f, under CAG treatment, stomatal apertures of WT, *plc1*, *gpa1*, and *plc1/gpa1* were inhibited 38.9%, 13.6%, 29.5%, and 14.1%, the stomatal apertures of *plc1* and *plc1/gpa1* were no significant difference, indicating that *PLC1* acts downstream of *GPA1*. In the *plc1* deletion mutant, the gene expression of *GPA1* was not significantly different from that of WT. However, in the *gpa1* deletion mutant, the research found that the gene expression of *PLC1* was significantly lower than that of WT, which further suggests that PLC1 was activated by GPA1 and then involved in CAG-induced stomatal movement (Fig. 3g, h).

### NIA1-and NOA1-dependent NO was essential for CAG-induced stomatal movement

**Fig. 4:**
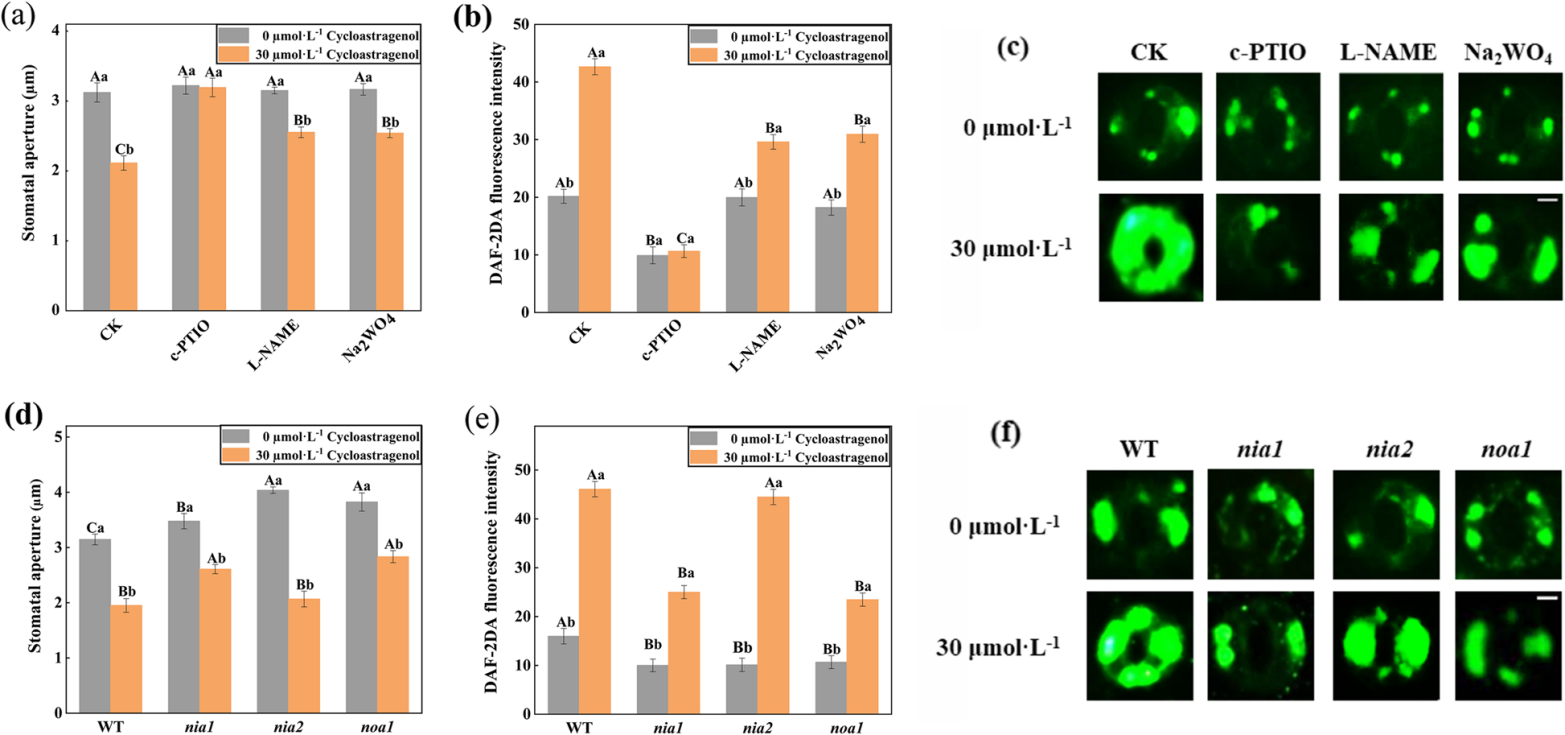
NIA1 and NOA1-dependent NO were essential for CAG-induced stomatal movement in *A. thaliana*. **a** After exogenous c-PTIO, L-NAME and Na_2_WO_4_ were applied, the stomatal apertures of *A. thaliana* leaves under the CAG treatment in WT. **b** and **c** Under different treatments, 10 μM fluorescent dye DAF-2DA was added in the dark for 15 min to measure the fluorescence intensity of NO in guard cells, and the stomatal images were taken. Bar = 2 μm. **d** Stomatal apertures of *A. thaliana* under the CAG treatment in WT, *nia1*, *nia2*, and *noa1*. **e** and **f** Under CAG treatment, the fluorescence intensity of NO in WT, *nia1*, *nia2*, *noa1*, and the stomatal images were taken. Bar = 2 μm. Uppercase letters indicate differences between groups, lowercase letters indicate differences within groups. Means with different letters are significantly different at *P* < 0.05

As shown in Fig. 4a, 30 µmol·L^−1^ CAG treatment significantly induced the stomatal closure of *A. thaliana*. The stomatal apertures induced by CAG increased by 51.10%, 20.79%, and 20.23% under 200 μM 2-(4-carboxyphenyl)-4, 4, 5, 5-tetramethylimidazoline-1-oxyl-3-oxide potassium (c-PTIO, an NO scavenger), 100 μM N^G^-nitro-L-Arg methylester (L-NAME, a specific inhibitor of NO synthase) and 100 μM tungstate (Na_2_WO_4_, an inhibitor of NR) treatments (Fig. 4a). The stomatal closure induced by CAG was completely blocked in the presence of c-PTIO, and the stomatal closure was partially blocked in the presence of L-NAME and Na_2_WO_4_, (Fig. 4a). These indicated that NO participated in the stomatal movement induced by CAG. Then, the fluorescence intensity of NO in guard cells was measured by fluorescent dye DAF-2DA. It was found that the green fluorescence intensity increased significantly after CAG treatment. NO was eliminated by 75.04% in the presence of 200 μM c-PTIO, and the green fluorescence intensity induced by CAG was partially inhibited by 30.59% and 27.47% in the presence of 100 μM L-NAME and 100 μM Na_2_WO_4_ (Fig. 4b, c). These indicated that CAG regulated stomatal movement by inducing NO accumulation. NOS and NR were responsible for the CAG-induced NO production.

**Fig. 5:**
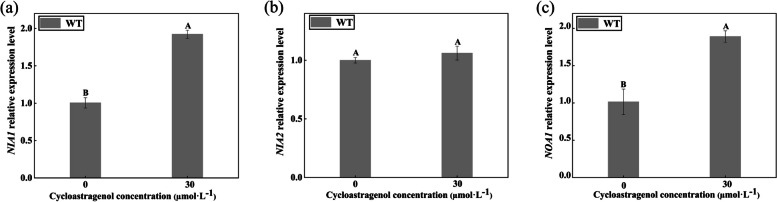
Gene relative expressions under the CAG treatment in WT. **a ***NIA1* relative expression under the CAG. **b ***NIA2* relative expression under the CAG. **c ***NOA1* relative expression under the CAG. Uppercase letters indicate differences between groups. Means with different letters are significantly different at *P* < 0.05

To further determine that NO participated in CAG-induced stomatal movement, stomatal apertures and CAG-induced NO production were detected in *nia1* and *nia2*, *noa1*. As shown in Fig. 4d, under CAG treatment, the stomatal apertures of WT were suppressed by 37.96%, and stomatal apertures of *nia2* were similar to WT. But, under the treatment of CAG, the degree of stomatal apertures of *nia1* and *noa1* was significantly lower than WT, which were suppressed by 25.01% and 25.87%, respectively. These indicate that NIA1 and NOA1 are involved in the stomatal movement induced by CAG. Similarly, CAG significantly stimulated the NO accumulation in WT guard cells, but the fluorescence intensity of NO in *nia1* and *noa1* was significantly lower than in WT (Fig. 4e, f). qRT-PCR analysis revealed that the gene relative expression levels of *NIA1* and *NOA1* were significantly increased by 47.74% and 46.33 under CAG treatment (Fig. 5). However, the gene relative expression levels of *NIA2* increased by only 5.76% under CAG treatment (Fig. 5). These results suggested that NIA1- and NOA1-dependent NO was essential for CAG-induced stomatal movement in *A. thaliana*.

### PLC1 acted on the upstream of NO to regulate the stomatal movement induced by CAG

**Fig. 6:**
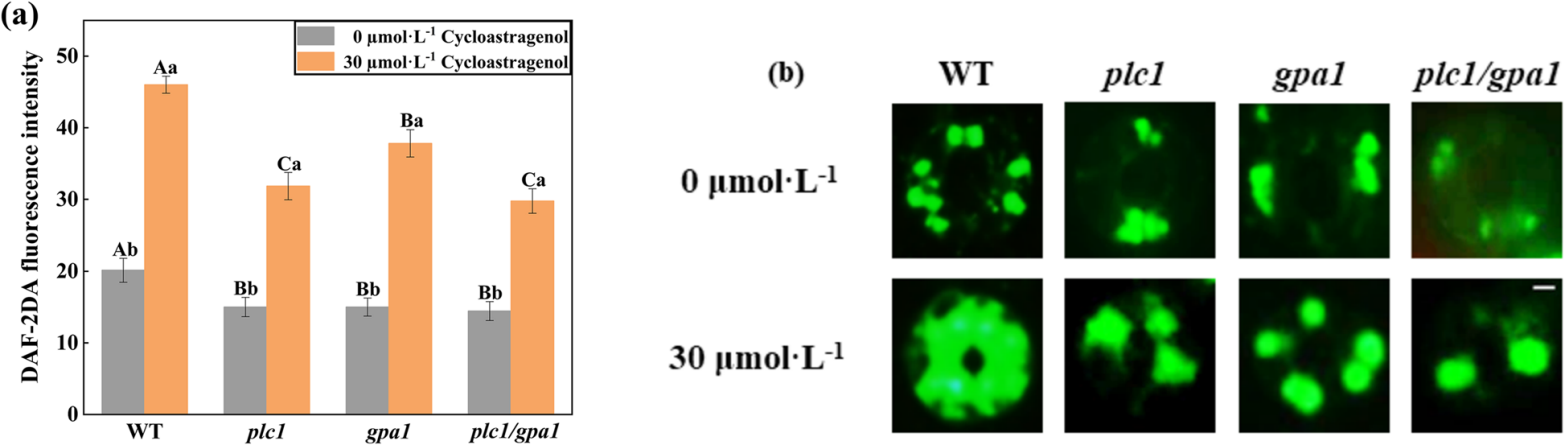
PLC1 and GPA1 positively regulate NO accumulation in guard cells under CAG treatment. **a** and **b** Under CAG treatment, 10 μM fluorescent dye DAF-2DA was added in the dark for 15 min to measure the fluorescence intensity of NO in WT, *plc1*, *gpa1*, and *plc1/gpa1*, and the stomatal images were taken. Bar = 2 μm. Uppercase letters indicate differences between groups, lowercase letters indicate differences within groups. Means with different letters are significantly different at *P* < 0.05

Previous studies have proved that PLC1 and NO are involved in the stomatal movement induced by CAG, but the relationship between them is unclear. Next, the NO production induced by CAG was detected in WT, *plc1*, *gpa1*, and *plc1/gpa1*. As shown in Fig. 6, the fluorescence intensity of NO in guard cells of *plc1*, *gpa1*, *plc1/gpa1* significantly increased under CAG, but was significantly lower than WT, indicating that PLC1 and GPA1 mediated the production of NO in guard cells. The results showed that PLC1 and GPA1 positively regulate NO accumulation in guard cells under CAG treatment. In addition, the content of NO in *plc1* and *gpa1* guard cells was lower than that in WT under CAG (Fig. 6). And stomatal apertures in *plc1* and *gpa1* were larger than that of WT (Fig. 3f). These results indicated that NO played a positive regulatory role in stomatal movement induced by CAG.

**Fig. 7:**
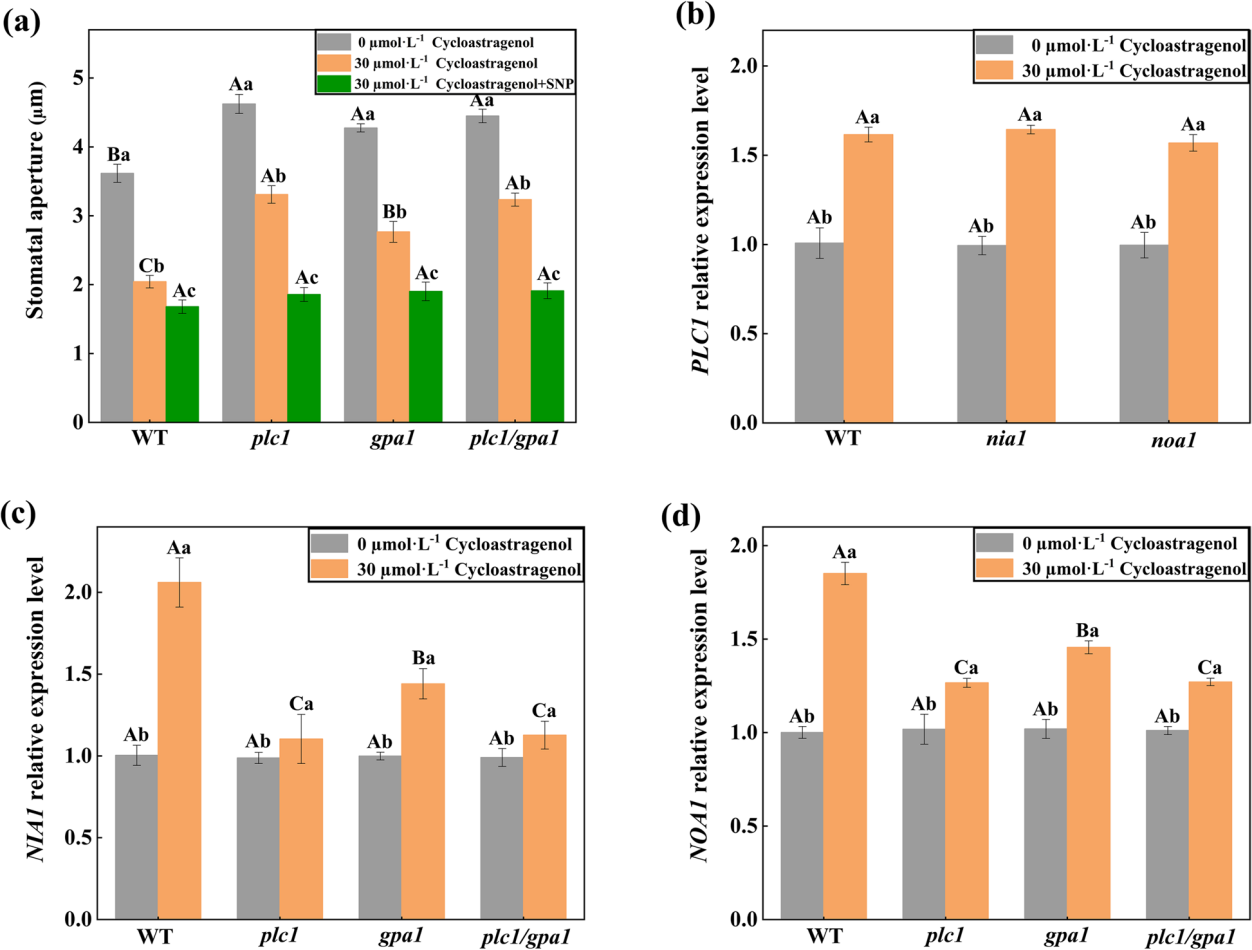
PLC1 and GPA1 located upstream of NO to regulate CAG-induced stomatal movement. **a** After 10 μM NO donor SNP was applied, stomatal apertures of *A. thaliana* leaves under the CAG treatment in WT, *plc1*, *gpa1*, and *plc1/gpa1*. **b** Relative expression level of PLC1 under the CAG treatment in WT, *nia1*, *noa1*. **c** Relative expression level of NIA1 under the CAG treatment in WT, *plc1*, *gpa1*, *plc1/gpa1*. **d** Relative expression level of NOA1 under the CAG treatment in WT, *plc1*, *gpa1*, *plc1/gpa1*. Uppercase letters indicate differences between groups, lowercase letters indicate differences within groups. Means with different letters are significantly different at *P* < 0.05

Exogenous nitric oxide donor Sodium Nitroprusside (SNP) significantly promoted stomatal closure in all the lines under CAG treatment (Fig. 7a). Under CAG treatment, the gene expression of *PLC1* in *nia1* and *noa1* was not significantly different from WT (Fig. 7b). But the gene expression levels of *NIA1* and *NOA1* in *plc1*, *gpa1*, *plc1/gpa1* decreased compared with WT (Fig. 7c, d). These implied that the accumulation of NO was essential for the CAG-induced stomatal movement, and PLC1 and GPA1 acted upstream of NO.

## Discussion

In recent years, with the rapid development of modern biological theory and technology and the increasing mutual penetration between different disciplines, allelopathy has gradually become a research hotspot. Allelochemical extract of *Moringa oleifera* promoted the growth of *Lepidium sativum* at the lowest tested concentration, while the highest tested concentration inhibited the growth of *Lepidium sativum* [28]. The study has also shown that parthenin from *Parthenium hysterophorus L*. showed an allelopathic effect by stimulating the growth of other plant species at low concentrations and suppressing the growth at high concentrations [29]. Crude extracts of different tissues from *Astragalus* plants have been reported to inhibit seed germination, radical elongation, and seedling growth of many other plants [30]. In this study, CAG affects the growth of *A. thaliana* (Fig. 1). Stomatal apertures decreased significantly after 30 µmol·L^−1^ CAG treatment for 1.5 h in *A. thaliana* (Fig. 2). These reflected that *A. thaliana* could respond to the influence of allelopathic substances by controlling the stomatal aperture.

Phospholipase C is a crucial hydrolase related to many physiological and anti-stress functions of plants. According to reports, PLC participates in many cell development and signal transduction pathways responding to abiotic and biotic stresses such as drought, high salt, low temperature, and high temperature [31]. PLC1 mediated the low-promotion and high-inhibition effect of Allelochemical Oridonin on the root growth in *A. thaliana* [32]. In this study, to explore the function of PLC1 in stomatal movement induced by CAG, the transgenic strains of *PLC1* were obtained (Figs. S2-S6). The increase of PLC activity and PLC1 gene expression positively correlated with CAG treatment (Fig. 3a, b), and the stomatal apertures of *plc1* were significantly different from WT (Fig. 3c). This further showed that PLC1 was involved in the allelopathy of CAG on *A. thaliana* stomatal movement.

GPA1 plays a very important role in stomatal movement. Stomatal closure induced by hydrogen-rich water depends on GPA1 in *A. thaliana* [33]. The results showed that the increase of GTP hydrolysis activity and GPA1 gene relative expression levels positively correlated with CAG treatment (Fig. 3d, e). In addition, preliminary work in our laboratory proposed that GPA1 is involved in the process of Oridonin-induced stomatal closure in *A. thaliana* and plays a role in the upstream of phospholipase Dα1 [21]. GPA1 and phospholipase Dδ (PLDδ) are required for JA-mediated regulation of osmotic resistance and seed germination [34]. In this study, to explore the function of PLC1 and GPA1 in stomatal movement induced by CAG, the homozygous double mutant of *plc1/gpa1* was obtained by hybridization and screening (Fig. S1). These demonstrated that PLC1 and GPA1 were involved in stomatal closure induced by CAG, and PLC1 acted downstream of GPA1 (Fig. 3f, g, h).

As a vital intermediate signal molecule in plants, NO participates in stomatal movement induced by many plant hormones and external environmental stimuli [35, 36]. NO improved the passive effects of p-hydroxybenzoic acid (pHBA) and (namely vanillic acid) VA by increasing PAL activity and enhancing the contents of antioxidative secondary metabolites [37]. NO is mainly synthesized by nitric oxide synthase (NOS) and nitrate reductase (NR). To investigate whether NO is involved in the CAG-induced stomatal movement, the effects of c-PTIO, L-NAME, and Na_2_WO_4_ and the NO content were analyzed. In this study, CAG-induced stomatal movement is inhibited by c-PTIO, L-NAME, and Na_2_WO_4_, which indicates that NO is involved in this process (Fig. 4a-c). NIA1 and NIA2 are involved in exogenous salicylic acid-induced NO generation and stomatal closure in *A. thaliana* [38]. In this study, under CAG treatment, the degree of stomatal closure of *nia1* and *noa1* was lower than WT, and the content of NO was also obviously lower than WT (Fig. 4d-f). The gene expression of *NIA1* and *NOA1* was significantly increased under CAG treatment (Fig. 5). These indicated that the NO from *NIA1* and *NOA1* is necessary for CAG to induce stomatal movement.

Preliminary research found that under drought stress, PLDδ mainly promoted the seed germination of *A. thaliana* through NO produced by the NR2 pathway [39]. Further studies indicate that GPA1 mediates several stimuli-regulated stomatal movements by inducing NO production in guard cells [40]. In this study, the NO fluorescence intensity in guard cells of *plc1*, *gpa1*, *plc1/gpa1* was lower than WT under CAG treatment (Fig. 6), and exogenous SNP significantly promoted stomatal closure in all the lines under CAG treatment (Fig. 7a). These illustrated PLC1 and GPA1 located upstream of NO to regulate CAG-induced stomatal movement.

**Fig. 8:**
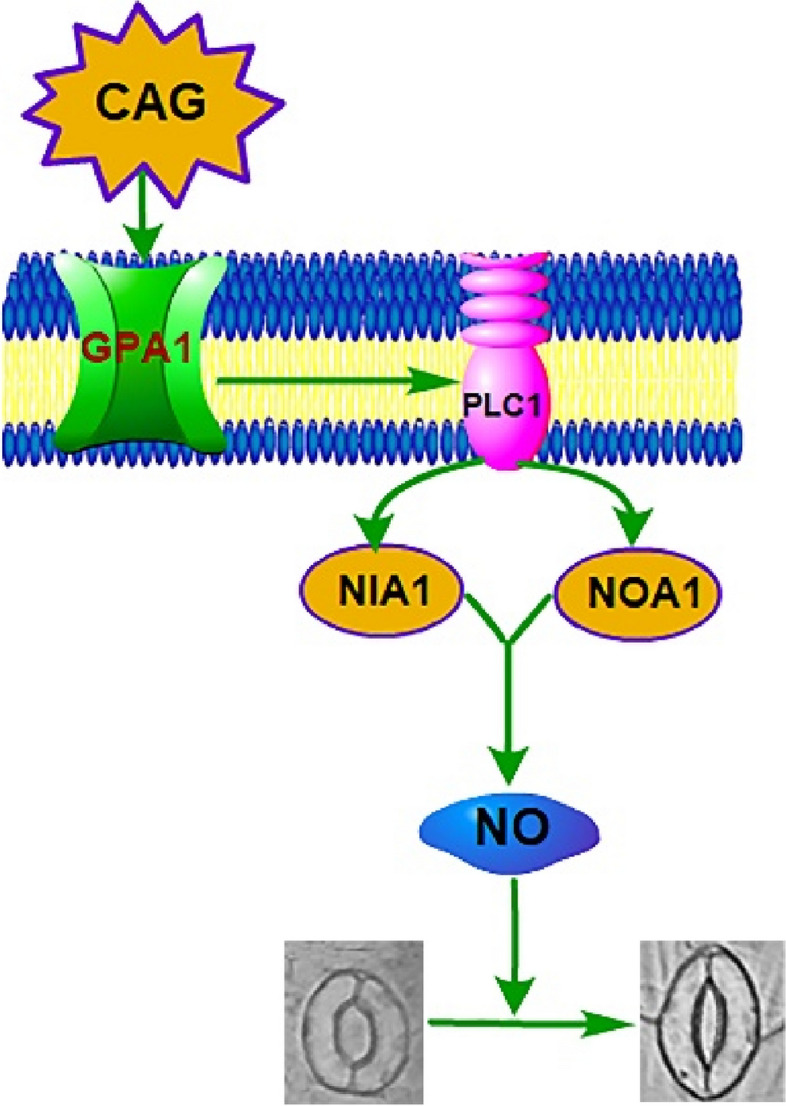
A schematic model of PLC1 and GPA1 involved in the process of CAG-induced stomatal closure in *A. thaliana*. Arrow end, activation

In summary, exploring the principles and influencing factors of stomatal movement is of great significance for clarifying how plants can minimize damage by adjusting the stomatal aperture under adversity conditions. Combining CAG with stomatal research for the first time, the study of the molecular mechanisms of CAG in stomatal research will contribute to the cultivation of drought-prone plants, as well as to weed control and the improvement of agricultural yields. The triterpenoid CAG at a concentration of 30 μmol·L^−1^ was able to significantly induce stomatal aperture reduction in *A. thaliana*. *PLC1* and *GPA1* were involved in CAG-induced stomatal movement, with *PLC1* acting downstream of *GPA1*. CAG induced stomatal movement by inducing the expression of *NIA1* and *NOA1* to accumulate NO in guard cells further. CAG activated PLC1 by affecting GPA1, thereby inducing the accumulation of NO and finally inducing stomatal movement (Fig. 8).

## Materials and methods

### Plant materials and treatments

All of the *Arabidopsis thaliana* mutants and transgenics employed shared the same (Col-0) genetic background. The T-DNA insertion mutants of *plc1* (SALK_025769c), *gpa1* (SALK_001846), *noa1* (CS6511), *nia1* (CS6936), *nia2* (CS2355) were obtained by the Arabidopsis Biological Resource Center (https://abrc.osu.edu). Cycloastragenol was purchased from Shanghai Shifeng Biological Co., Ltd., with a purity of ≥ 98%.

The *A. thaliana* seeds were cultured according to the study with minor modifications [41]. Seeds were sterilised with 75% ethanol and 0.5% NaClO for approximately 15 s and then washed three times with sterile water. Surface-sterilized seeds were sown on 1/2 MS medium (3% sucrose, 0.6% agar, pH 5.8). Afterward, the plates were incubated in a growth chamber under a 16 h light/8 h dark photoperiod at 22 ℃. For pharmacological treatments, Cycloastragenol was dissolved in DMSO, and added to the medium at 30 µM.

### Generation of transgenic plants

Transgenic plants were generated according to the study with minor modifications [41]. For the generation of *PLC1* complementation lines and overexpressed lines, full-length cDNA of *PLC1* was amplified using by high-fidelity DNA polymerase (Prime STAR HS DNA Polymerase, Clontech) and subcloned into p-DONR vector. Confirmed construct was transformed into Escherichia coli and *PLC1* was cloned into the destination vector pBIB-35S-*PLC1*-GFP vector via LR reaction. After verification of the construct by using traditional Sanger sequencing, the construct was transformed to *plc1* mutant and WT via an Agrobacterium tumefaciens-mediated floral-dip method.

### Determinations of PLC activity and GTP activity

PLC activity were measured according to the study with minor modifications [42, 43]. 0.2 g plant material were ground to a homogenate on ice with 1.8 mL 0.01 mol·L^−1^ PBS (pH 7.4), and centrifuged at 4000 rpm for 15 min at 4 ℃. The supernatant was used for assay. PLC activity measurement was performed using the PLC assay kit (Shanghai MLBIO Biotechnology Co. Ltd., China) according to the description of the manual.

GTP hydrolysis activity was measured as described previously [41]. Briefly, the homogenate was isolated from 0.2 g *A. thaliana* seedlings by 1.8 mL 0.01 mol·L^−1^ PBS (pH 7.4). The homogenate was centrifuged at 4000 r·min^−1^ for 15 min at 4 °C. The GTP hydrolysis activity was detected by a GTP hydrolysis assay kit (Shanghai MLBIO Biotechnology Co. Ltd., China). The manipulation followed the protocol of the GTP hydrolysis assay kit.

### RNA extraction and Real-time quantitative PCR (qRT-PCR) analysis

Total RNA extraction, reverse transcription PCR and qRT-PCR analysis were measured as described previously with minor modifications [41]. Total RNAs were isolated using an RNAiso Plus Kit (Takara, Shiga, Japan). For transcript level analysis, cDNAs were synthesized using a cDNA Synthesis kit (Takara, Tokyo, Japan). qRT-PCR analysis was performed with the SYBR® Premix Ex Taq kit (Takara, Tokyo, Japan) using the IQ5 Multicolor Real-time PCR Detection System, and ACTIN gene was used as an internal standard. The cycling conditions were Cycle 1 (1×): 95.0 °C. for 10 min; Cycle 2 (40×): 95.0 °C. for 15 s, 60.0 °C. for 30 s; Cycle 3 (81×): 72.0 °C. for 30 s. The qRT-PCR primers are given in Table S1.

### Detection and quantification of NO

NO production was detected by specific fluorescent probes 4,5-diaminofluorescein diacetate (DAF‐2 DA; Cayman Chemical, USA) [44]. Epidermal strips of treated leaves were incubated in Tris-HCl buffer containing 10 µM DAF-2 DA in the dark for 15 min. Afterward, the leaves were rinsed more than three times in Tris-HCl buffer to wash away the excess fluorescent dye. They were detected and photographed using a confocal microscopy (Leica DM4000B, Germany). Fluorescence density was analyzed on Imag-Pro Plus software.

### Stomatal bioassays

Leaves were selected from the same position and the similar size at the rosette leaves for stomatal aperture measurements. Stomatal apertures were measured as described previously with minor modifications [45, 46]. In brief, freshly 3-week-old *Arabidopsis* seedlings were incubated in MES-KCl buffer (50 mM KCl, 10 mM MES and 50 mM CaCl_2_, pH 6.15) under light for 2 h to induce stomatal opening. Subsequently, incubated in MES-KCl buffer alone or MES-KCl buffer containing 30 µM Cycloastragenol, 200 µM c-PTIO, 100 µM L-NAME or 100 µM Na_2_WO_4_ under the same light conditions for 30 min-2 h. Subsequently, the abaxial epidermis of *Arabidopsis thaliana* was placed onto a slide and photographs were taken using the Leica DM4000B. The aperture width of each stomatal pore was determined from the image. To avoid the potential effects of the circadian rhythm on stomatal aperture, stomatal assays were always started at the same time of day. In each experiment, 30 pairs of guard cells of each plant line were measured, and three replications were maintained for each stomatal assay experiment. Stomatal apertures were digitized using a Dn-3 Image Analysis System (Ningbo, China). The difference at the level of *P* < 0.05 was regarded as significant.

### Statistical analysis

Three technical replicates were performed for each experiment. The data showed as the mean ± standard error (SE). The statistically significant differences were analyzed with SPSS version 17.0, and error bars were determined based on Duncan’s multiple range test. *P*-value < 0.05 was considered statistically significant. Origin 9.0 drawing software was used for drawing.

### Supplementary Information

**Additional file 1: Fig. S1.** Identification of *plc1/gpa1* double mutant. Molecular analysis of WT, *plc1*, *gpa1*, and *plc1/gpa1*, primers LP, RP and LB (LBb1.3) were used to target the flanking sequences of the T-DNA. **Fig. S2.** PCR amplification of CDS of *PLC1*. **Fig. S3.** Construction of *35S-AtPLC1-GFP* recombinant vector. **Fig. S4.** Screening of *PLC1* transgenic trains. **Fig. S5.** PCR results of generation seedings. **Fig. S6.** RT-qPCR results of transgenic. **Table S1.** List of gene primers for qRT-PCR. **Table S2.** List of primers used for PCR identification.

## Data Availability

All study data are included in the manuscript and its additional files.

## Abbreviations

CAG: Cycloastragenol
PLC1: Phospholipase C1
GPA1: Gα-submit of the heterotrimeric G-protein
NIA1: Nitrate reductase 1
NOA1: Nitric oxide-associated 1
NO: Nitric oxide
c-PTIO: 2-(4-Carboxyphenyl)-4, 4, 5, 5-tetramethylimidazoline-1-oxyl-3-oxide potassium
L-NAME: NG-nitro-L-Arg methylester
*A. thaliana*: *Arabidopsis thaliana*
