# Supplementary material for: PLC1 mediated Cycloastragenol-induced stomatal movement by regulating the production of NO in Arabidopsis thaliana
Source: BMC Plant Biol. 2023 Nov 17;23:571. doi: 10.1186/s12870-023-04555-7 (PMC10655312; doi:10.1186/s12870-023-04555-7)
Supplement: Supplementary file 1 — Additional file 1: Fig. S1. Identification of plc1/gpa1 double mutant. Molecular analysis of WT, plc1, gpa1, and plc1/gpa1, primers LP, RP and LB (LBb1.3) were used to target the flanking sequences of the T-DNA. Fig. S2. PCR amplification of CDS of PLC1. Fig. S3. Construction of 35S-AtPLC1-GFP recombinant vector. Fig. S4. Screening of PLC1 transgenic trains. Fig. S5. PCR results of generation seedings. Fig. S6. RT-qPCR results of transgenic. Table S1. List of gene primers for qRT-PCR. Table S2. List of primers used for PCR identification. [file 12870_2023_4555_MOESM1_ESM.docx]

**PLC1 mediated Cycloastragenol-induced stomatal movement by regulating the production of NO in *Arabidopsis thaliana***

Juantao Kong, Rongshan Chen, Ruirui Liu, Wei Wang, Simin Wang, Jinping Zhang, Ning Yang*

College of Life Science, Northwest Normal University, Lanzhou 730070, China

*Correspondence: Ning Yang (xbsd-yn@163.com)

**Supplementary Material**

**Identification of the *plc1*, *gpa1* and *plc1/gpa1* mutants**

To obtain the homozygosis strains of *plc1*, *gpa1*, and *plc1/gpa1*, seeds were sown and cultured for 4 weeks, then DNA was extracted using the Easy Pure Plant Genomic DNA Kit. The primers were designed according to https://signal.salk.edu/tdnaprimers.2.htmL (Table S1). With WT as control, the mutant was identified by the ‘three-primer method’. The homozygous *plc1* and *gpa1* can be amplified with T-DNA specific primers (LBb1.3+RP). In subsequent experiments, using the hybridized *plc1* and *gpa1* as the parents, the *plc1/gpa1* double mutants were identified and screened (Fig. S1a, d). Compared with the WT, the gene relative expression levels of PLC1 and GPA1 were weak in *plc1* and *gpa1*, respectively. And the relative gene expression levels of PLC1 and GPA1 in *plc1/gpa1* were also very weak. The PLC activity and GTP hydrolase activity in *plc1*, *gpa1*, and *plc1/gpa1* mutants were significantly lower than those in the WT (Fig. S1b, c, e, f). In summary, we successfully constructed homozygous *plc1/gpa1* double mutant, which can be used in subsequent experiments.


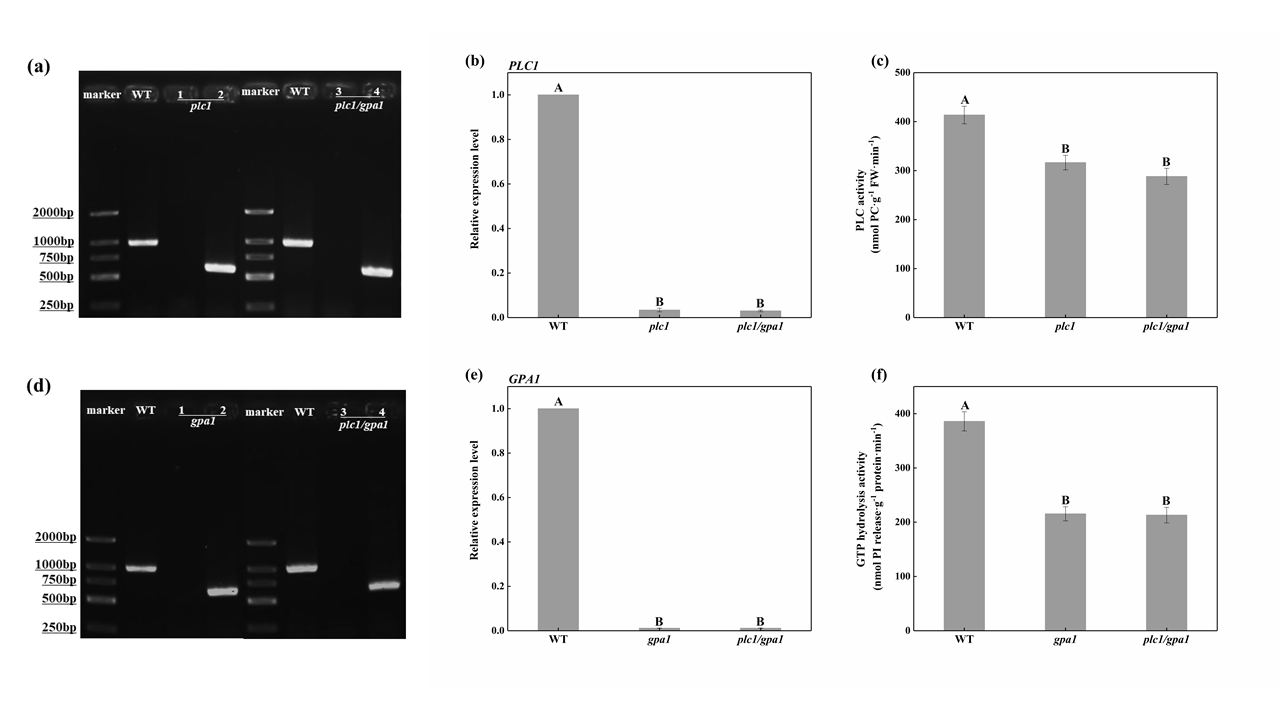


Fig. S1 Identification of *plc1/gpa1* double mutant. Molecular analysis of WT, *plc1*, *gpa1*, and *plc1/gpa1*, primers LP, RP and LB (LBb1.3) were used to target the flanking sequences of the T-DNA. (a) Lanes are primers for AtPLC1, lane 1 and lane 3: template + AtPLC1LP + AtPLC1RP, lane 2 and lane 4: template + LBb1.3 + AtPLC1RP. (b) qRT-PCR analysis of PLC1 relative expressions in WT, *plc1*, *plc1/gpa1*. (c) The PLC activity in WT, *plc1*, *plc1/gpa1*. (d) Lanes are primers for AtGPA1, lane 1 and lane 3: template + AtGPA1LP + AtGPA1RP, lane 2 and lane 4: template + LBb1.3 + AtGPA1RP. (e) qRT-PCR analysis of GPA1 relative expressions in WT, *gpa1*, *plc1/gpa1*. (f) The GTP hydrolysis activity in WT, *gpa1*, *plc1/gpa1*. Uppercase letters indicate differences between groups. Means with
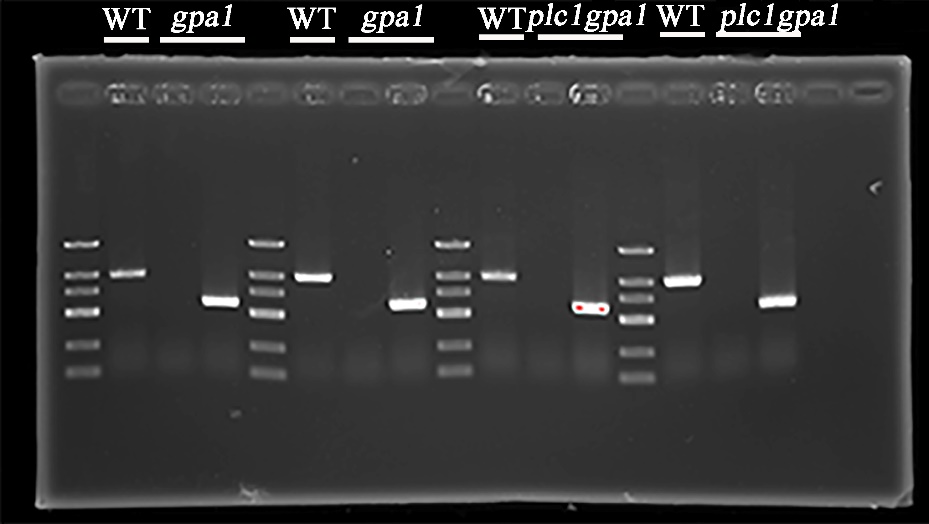

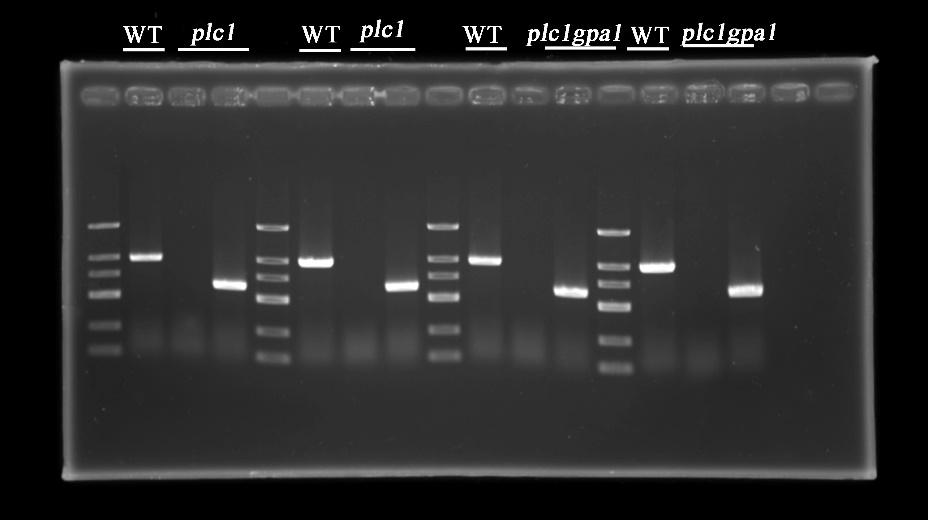
different letters are significantly different at P<0.05.

Identification of *plc1/gpa1* double mutant（Original picture）

**Generation of transgenic plants**


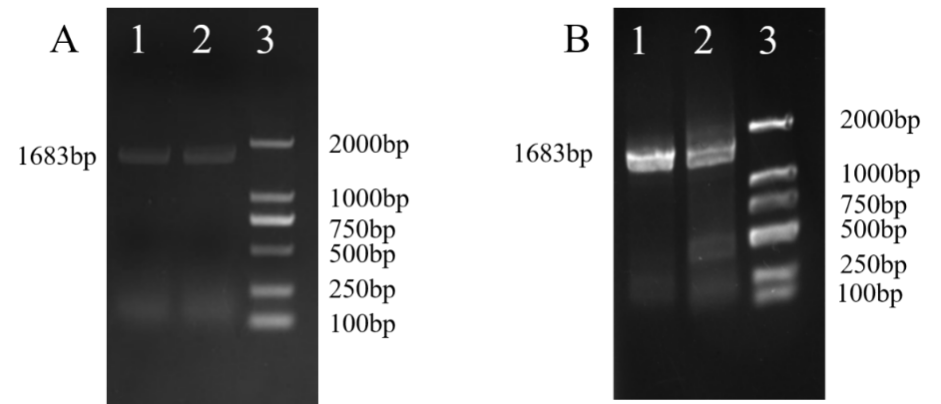
To generate *PLC1* complementation lines and overexpressed lines, the genomic sequence of *PLC1* containing 1683 bp cDNA sequence was amplified by high-fidelity DNA polymerase (Prime STAR HS DNA Polymerase, Clontech). The PCR product was first introduced into the p-DONR vector (Invitrogen) by BP reaction and then was cloned into the destination vector pBIB-35S-*PLC1*-GFP vector via LR reaction. After verification of the construct by using traditional Sanger sequencing, the construct was transformed to *plc1* mutant and WT via an Agrobacterium tumefaciens-mediated floral-dip method.

Fig S2 PCR amplification of CDS of *PLC1*

(A) PCR amplification of CDS (B) PCR amplification of attB


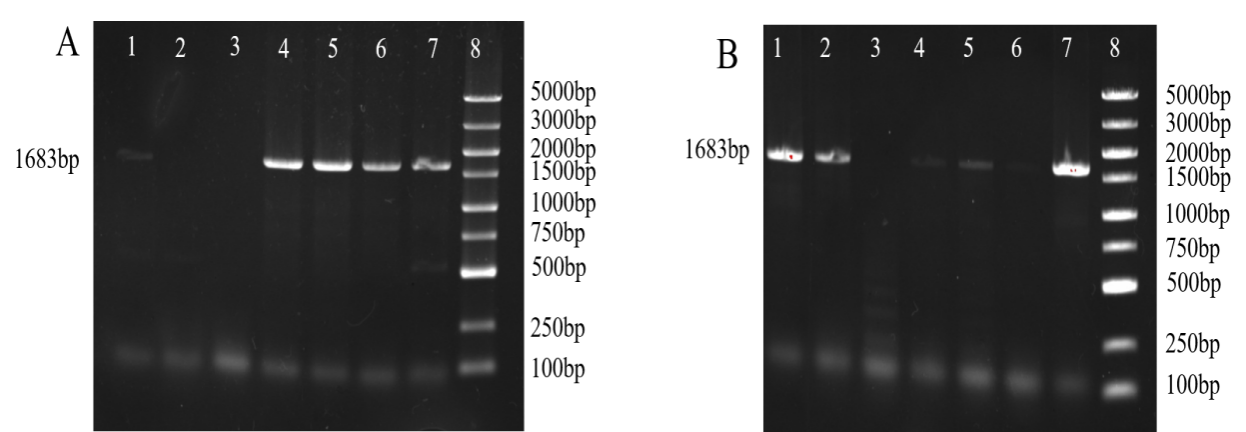


Fig S3 Construction of *35S-AtPLC1-GFP* recombinant vector

(A) Electrophoregram of bacterial fluid PCR results of BP

(B) Electrophoregram of bacterial fluid PCR results of LR


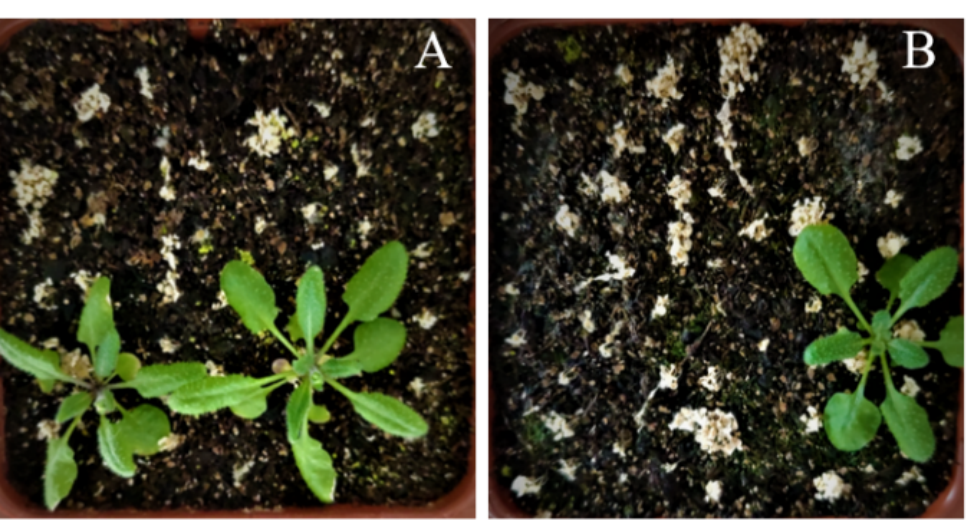


Fig S4 Screening of *PLC1* transgenic trains

(A) Screening of *PLC1-OE* line Basta

(B) Screening of *PLC1-*CO line Basta


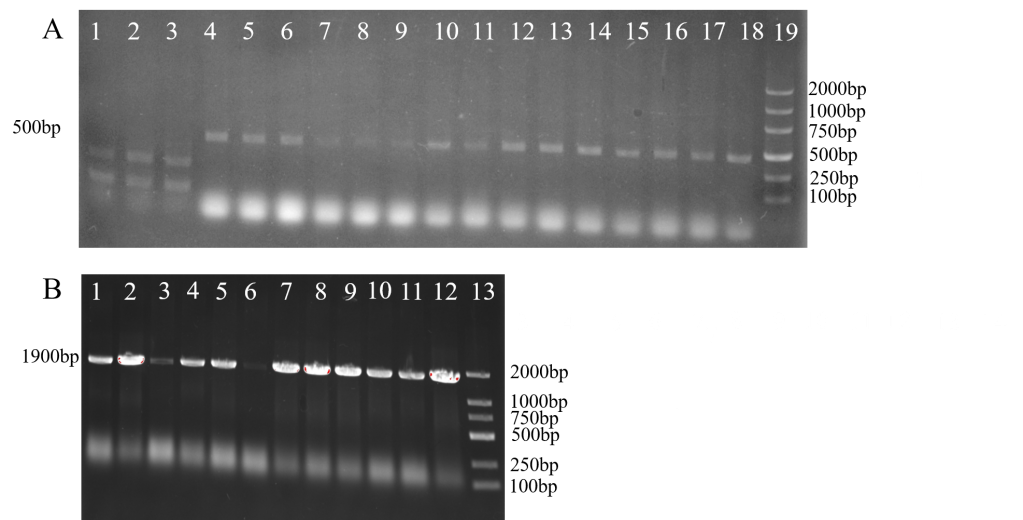


Fig S5 PCR results of generation seedings.

(A) DNA identification of *PLC1* overexpression strains;


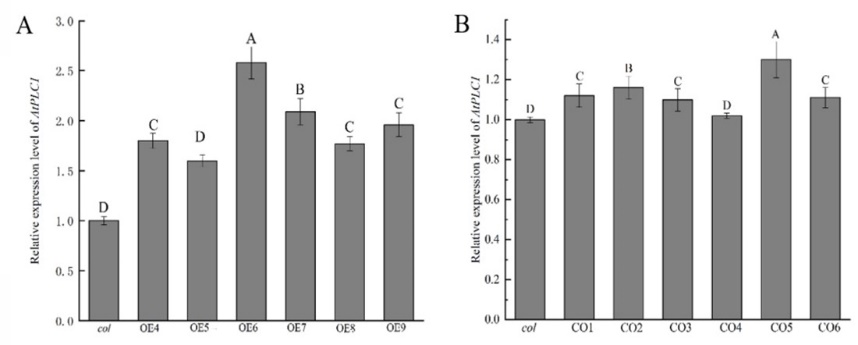
(B) DNA identification of *PLC1* complementary strains;

Fig S6 RT-qPCR results of transgenic

(A) *PLC1-OE* line

(B) *PLC1-*CO line

**Table S1** List of gene primers for qRT-PCR

| Primers | Gene sequence (5´-3´) |
| --- | --- |
| *Actin* FP | 5´-TGTGCCAATCTACGAGGGTT-3´ |
| *Actin* RP | 5´-TTTCCCGCTCTGCTGTTGT-3´ |
| *AtPLC1* FP | 5´-CTCAAAAGGTTCAACAACGGAT-3´ |
| *AtPLC1* RP | 5´-AGCGTGAATCGAGATTAAGTCT-3´ |
| *AtGPA1* FP | 5´-ACAGGCTGCTGAAATCGAAAGC-3´ |
| *AtGPA1* RP | 5´-TGGCATGAATGACTGGAACATAG-3´ |
| *AtNIA1* FP | 5´-AGCCTGGGACGAGTCTTTCAATAC-3´ |
| *AtNIA1* RP | 5´-CGGGTCTGGTCGGGTGTTC-3´ |
| *AtNIA2* FP | 5´-ATCATCATCCCCGGTTTCATTGGT-3´ |
| *AtNIA2* RP | 5´-GTCGGCGAGTTCGGCGTCT-3´ |
| *AtNOA1* FP | 5´-CCTTCTCTTCCTCGTCGCCACAC-3´ |
| *AtNOA1* RP | 5´-CGCCGCAAATCCATCTCGTTC-3´ |

**Table S2** List of primers used for PCR identifcation

| Primers | Gene sequence (5´-3´) |
| --- | --- |
| LBb1.3 | 5´-ATTTTGCCGATTTCGGAAC-3´ |
| *AtPLC1* LP | 5´-CGATTGGGAGATCAGGAG-3´ |
| *AtPLC1* RP | 5´-AAATGGTGGTAGGCGTGT-3´ |
| *AtGPA1* LP | 5´-TAAAGCTTCGTTTATGCAGCC-3´ |
| *PLC1CDS* F | 5’-ATGAAAGAATCATTCAAAGTGTGTTTCTG-3’ |
| *PLC1CDS* R | 5’-ACGAGGCTCCAAGACAAACCGCATG -3’ |
| *PLC1pro* F  *PLC1pro* R | 5’-CGATTGGGAGATCAGGAG-3’  5’-AAATGGTGGTAGGCGTGT -3’ |
| *PLC-GFP*-F  *PLC-GFP*-R | 5’-TTCTTAGTGATTTCAA-3’  5’-ACCCCCATCGGCGAC -3’ |

※ LP, RP represent the upstream primer and downstream primer
